# Contributions to More Sustainable and Climate-Resilient Cattle Production: Study of Performance of Galloway and Highland Breeds in Transylvania, Romania

**DOI:** 10.3390/ani14243686

**Published:** 2024-12-20

**Authors:** Mirela Ranta, Anamaria Mălinaș

**Affiliations:** 1 Department of Plant Culture, Faculty of Agriculture, University of Agricultural Sciences and Veterinary Medicine, Calea Mănăştur 3-5, 400372 Cluj-Napoca, Romania; mirela.ranta@usamvcluj.ro; 2 Department of Environmental Protection and Engineering, Faculty of Agriculture, University of Agricultural Sciences and Veterinary Medicine, Calea Mănăştur 3-5, 400372 Cluj-Napoca, Romania

**Keywords:** Galloway calves, Highland calves, average daily gain (ADG), feed quality, performance, adaptability

## Abstract

Sustainable and climate-resilient livestock systems are increasingly necessary to balance food production demands with environmental conservation. Breeds such as Galloway (Ga) and Highland (Hi) cattle are recognized for their adaptability to extensive grazing systems, low input requirements, and ability to thrive on marginal lands. Despite their potential, research on the performance of Ga and Hi cattle in low-resource, extensive grazing systems, particularly in Romania, remains scarce. This study evaluated the performance of Ga and Hi beef cattle raised under low-input conditions with a focus on the following: (1) the average daily gain (ADG) on low- and medium-quality forage and (2) the cattle’s adaptability to extensive grazing systems. The study, conducted at Cojocna Farm, Transylvania, Romania (2023–2024), involved five male and three female calves from each breed. Calves were weighed five times in the entire observation period, and feed quality was analyzed. The results showed that Ga calves, especially males, had a higher ADG than Hi calves (Ga = 676.91 g, Hi = 581.14 g), while females showed more consistent performance during winter feeding. Both breeds demonstrated strong adaptability and satisfactory performance under the extensive conditions of Transylvania, as evidenced by the comparison of the obtained ADG with the values provided by the National Breed Register. Future research should explore the long-term sustainability of these breeds in varying environmental conditions, to investigate genetic factors influencing performance, and assess the broader ecological and economic benefits of integrating Galloway and Highland cattle into diverse farming systems.

## Simple Summary

The aim of this study was to evaluate the performance of Galloway (Ga) and Highland (Hi) cattle under low-input, extensive grazing conditions in Transylvania, Romania. The results indicate that both breeds show strong adaptability to these systems, with Ga calves, particularly males, exhibiting a higher average daily gain (ADG) compared to Hi calves. Additionally, this study highlighted that both breeds are resilient to the challenging environmental conditions, as demonstrated by their performance and a comparison with national breed averages. These findings suggest that Ga and Hi cattle, due to their ability to thrive on low-resource grazing lands, are valuable assets for sustainable and climate-resilient livestock systems. This research could be valuable for farmers, policy-makers, and agricultural stakeholders aiming to implement climate-resilient practices in regions with limited resources.

## 1. Introduction

Livestock farming plays an essential role in global agricultural systems, ranking second only to crop production in terms of economic value [1,2]. Furthermore, livestock contributes to community resilience and well-being by providing essential services such as income, transportation, draught power, and soil nutrients [3]. Beef cattle, in particular, are extremely important for global food security and economic stability, offering a high-quality protein source and supporting rural livelihoods, especially in regions where other food sources are less viable. Despite this significance, cattle production faces scrutiny for its greenhouse gas (GHG) emissions, accounting for about 41% of agriculture’s total emissions through methane (CH_4_) released by enteric fermentation and manure management; compounding this, climate change itself poses risks to cattle production, with shifting temperatures and unpredictable rainfall impacting traditional breeds and increasing production costs [4,5]. This dual challenge has driven interest in sustainable cattle management practices that could mitigate environmental impact and enhance resilience using cattle breeds like Highland (Hi) and Galloway (Ga) [6,7].

Sustainability in beef production relies on efficient farm practices, high productivity, and value chains that meet market specifications while minimizing environmental and animal welfare impacts [8,9]. Transitioning to lower-input systems is key to reducing environmental footprints, addressing resource limitations, and meeting the demand for quality while adapting to climate impacts. Studies emphasize that climate-resilient systems depend on selecting appropriate genotypes, as breeding herds that thrive under extensive systems can reduce feed costs, which make up over 60% of production expenses [1].

Grasslands and rangelands provide a sustainable nutrient source for low-impact beef production systems [10]. Given that these systems rely on breeds capable of converting forage into meat—even when forage quality is low—strategic breed selection is essential. With large areas of grasslands possessing low to medium forage quality, the strategic selection of cattle breeds becomes even more critical. While some breeds may underperform, resilient breeds like Highland (Hi) and Galloway (Ga) cattle can thrive, highlighting the importance of breed selection for maximizing production efficiency [11,12].

The Transylvanian Plateau, Romania, offers a unique setting for studying cattle breeds in extensive grazing systems. The region’s topography and climate bring both challenges and opportunities for sustainable livestock production. Historically, native cattle breeds such as the Bălțata românească Simmental type (or Romanian Spotted Cattle) have been integral to Transylvanian agriculture, but these breeds often struggle with lower productivity and limited adaptability to the changing climate conditions [13,14]. The increasing unpredictability of weather patterns, especially in the context of climate change, necessitates the exploration of more resilient cattle breeds. The Ga and Ha breeds, originating from the Scottish Highlands, are particularly well suited to extensive grazing systems in these harsh conditions. These breeds have shown an ability to thrive on low-quality forage, making them an ideal candidate for low-input farming systems [15]. Their resilience and adaptability to environmental stressors, such as cold temperatures and limited feed resources, make them promising for regions like Transylvania, where such grazing systems can be implemented sustainably.

Will the Highland and Galloway breeds be able to adapt and perform well if grazed on poor- to medium-quality grassland?

In this context, this study aims to assess the performance of Ga and Hi cattle in extensive pasture systems characterized by low- to medium-quality forage in Transylvania, Romania. Specifically, this study focuses on the following: (1) evaluating the average daily gain (ADG) of Ga and Hi cattle, fed with low- to medium-quality forage, and (2) investigating the adaptability of these breeds to low-input, extensive grazing systems. By analyzing the performance of these breeds under local conditions, this research aims to determine how they can contribute to sustainable and climate-resilient livestock farming, particularly in mitigating the environmental impacts of traditional cattle farming practices.

## 2. Materials and Methods

### 2.1. Study Site

This research was carried out at the Didactic and Experimental Station—Cojocna Farm—belonging to the University of Agricultural Sciences and Veterinary Medicine Cluj-Napoca, which is located in Cojocna, Cluj County, in the western part of the Transylvanian Plain, which is an integral part of the Transylvanian Depression. The geographical coordinates are 46°44′07′′ N 23°53′50′′ E with an average altitude of 350–500 m. The soil type is Gleissolized Cernisol, soil class Gleisols; the average annual temperature is 9.86 °C; and the annual sum of rainfall is 789.5 mm.

### 2.2. Animals

This experiment was conducted on Ga and Hi cattle in 2023 and 2024 with the following calves: 5 male and 3 female Ga calves and 5 male and 3 female Hi calves, for a total of 16 calves of both breeds.

The calves were weighed to determine the ADG; the weighing was carried out five times per calf using a cattle weighing stand, as follows: each calf was weighed for the first time on the day of birth (this weighing was based on the date of birth of the calves of each breed), the second weighing was carried out around the time of weaning in September 2023, the third weighing was carried out in November 2023, the fourth weighing was carried out in February 2024, and the fifth weighing was carried out in May 2024.

The following formula was used to determine the average daily gain:

$$ADG=\frac{Mf-Mi}{T}$$

Average daily gain (g): Mf—the final body mass (kg), after the fattening duration or control period; Mi—the starting body mass (kg) (at the beginning of the control period or at the beginning of the fattening period); T—the time or duration in days of the fattening or control period [16].

**Figure 1:**
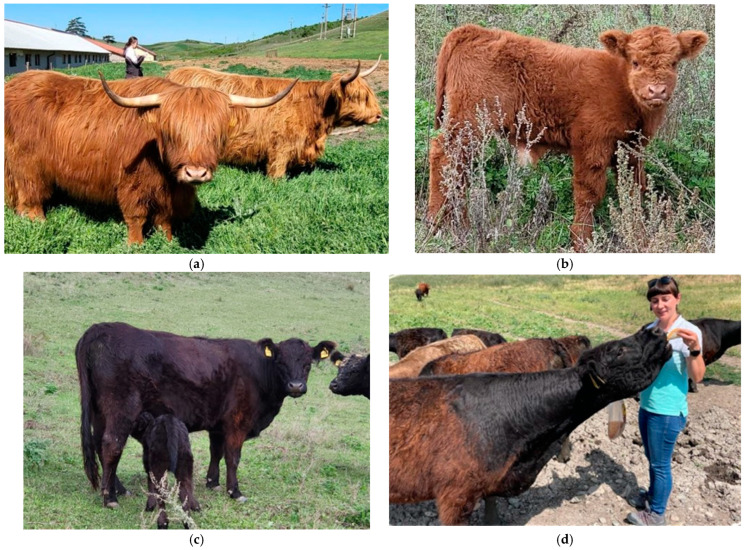
Representative images of original aspects documented during this study: (**a**) Highland—cows; (**b**) Highland—calf; (**c**) Galloway—mother with calf; (**d**) Galloway—cows.

This study is part of a project at the University of Agricultural Sciences and Veterinary Medicine Cluj-Napoca. The cows and bulls (for each breed) were purchased and imported from Germany, and all animals have certificates of origin. Throughout the project, we benefited from the technical expertise provided by the breed association in Germany. To assess the adaptability of the breed, only calves born in Cojocna, Romania, were used in this study. To maintain origin, all animals were registered in the National Breed Register (Figure 1).

### 2.3. Animal Feeding

This study was carried out at the Cojocna Farm (the area of the whole farm is 667 ha, of which 400 ha is arable land, and 267 ha consists of grassland and meadows).

The livestock of both breeds was extensively fed throughout the year. Each breed was allocated 18 ha of natural grassland of poor to medium quality (*Festuca rupicola* type and *Stipa capillata*–*Bothriochloa ischaemum* type [17]), where they grazed (April to October). The welfare of the animals was carefully monitored throughout this study. The animals included in this study received the required vaccinations (Anthrax), as well as internal and external parasite control treatments.

During the winter period (November–March), calves were fed with hay of poor to medium quality, available ad libitum. A shelter was available for the winter, but the calves preferred to eat and sleep outside due to the special coats of both breeds, which ensure thermal protection and water drainage. Water was provided both during the summer grazing period and in winter through constant level troughs, which were regularly cleaned and disinfected. In the winter, they had an antifreeze system. Salt was also available in both seasons. There was no supplementary feeding.

### 2.4. National Breed Register

Average daily gain data for the Ga and Hi breeds were also retrieved from the National Breed Register to compare them with data from the studied farm and to assess breed performance. The National Breed Register provided ADG data for calves collected across 41 counties in Romania; hence, these calves were fed differently and were exposed to different pedoclimatic conditions.

### 2.5. Feed Quality

The feed samples were collected in the field, both for grassland and hay, and the forage analysis was performed by the Laboratory of Applied Biological Sciences of the University of Agricultural Sciences and Veterinary Medicine Cluj-Napoca, Romania.

The protein content of the forages was determined using the Kjeldahl method, involving the digestion of the sample with sulfuric acid in the presence of catalysts. Crude fat content was analyzed using the Soxhlet method, in which total fat is extracted using light petroleum [18]. Crude fiber was assessed using the Weende method, in which non-cellulosic compounds are solubilized using sulfuric acid and potassium hydroxide solutions. NDF (neutral detergent fiber) and ADF (acid detergent fiber) contents were determined according to the procedure described by Van Soest [19].

### 2.6. Statistical Analysis

The distribution of the data sets (animal measurements) was tested for normality using the Shapiro–Wilk test (PAST 4.17, NHM, Oslo, Norway). Parametric tests were applied further. A two-way ANOVA was used to explore the influence of factors and their interaction on the studied parameters (α 0.05). Significant findings were further explored with Tukey’s test in order to identify differences between variants (OriginLab, Northampton, MA, SUA). Pearson’s correlation was used to study the relationship between variables, while a *t*-test (PAST 4.17, NHM, Oslo, Norway) was used to compare the mean experimental values with those of National Registry of Breed from Romania.

## 3. Results

### 3.1. Performance of Calves

A two-way ANOVA revealed that breed exerted a statistically significant influence on weighing. Furthermore, breed had a highly significant influence on the weight of calves at birth and in winter. Gender exerted a significant influence on weight in autumn (September, November) and spring (May). The bipartite interaction between breed and gender exerted a significant influence on the weight of calves in November (Table 1).

The parameters were analyzed in more detail; the average results of the five weighings on the changes in the weight of the calves taken in this study and the differences between genders are presented in Table 2.

For the Hi breed, the mean weight of calves at birth ranged from 25.60 kg for male calves to 23.33 kg for female calves. The means of the first weighing did not show significant differences between genders (Table 2). For the Ga breed, the mean birth weight of calves was higher than that for Hi and ranged from 30.00 kg for male calves to 26.33 kg for female calves. For this breed, male calves had significantly higher weight values than females (Table 2).

At birth, Ga males were statistically significantly superior and remained significantly superior in September. Although Hi males were statistically significantly superior to Hi females at birth, the difference was not statistically significant.

The dispersion indices (standard deviation) for the Hi breed show that in November, the standard deviation for females was lower (2.08 kg) than that for males, indicating greater uniformity among female calves. In contrast to the Hi breed, the Ga breed had a higher standard deviation for both males and females. However, females had a lower standard deviation for both breeds. In February, the standard deviation for females of both breeds became much more similar (no extremes in weight). In May, Hi females remained uniform with lower standard deviations than Hi males and Ga males and females.

In regard to ADG, although in autumn (September and November) there are differences in the values of the experimental variants, the differences are not statistically significant, which can be explained by the large standard deviations recorded (Table 3).

In February and May, there are statistically significant differences for ADG, with Ga males having higher values. In May, Ga males retain higher ADG values, but they are not statistically significant compared to Hi males or Ga females, but it is worth noting that the females of both breeds had lower standard deviations than males in February (the period in which they were fed hay), indicating that they responded more uniformly to hay feeding than males, whose standard deviation for ADG only started to decrease in May after they were put out to pasture.

The average total gain for the Hi breed was 581.14 g for males and 583.90 g for females, and for the Ga breed, it was higher for males at 676.91 g and 644.33 g for females.

Because the influence of breed exerted a significant effect on ADG, differences between breeds were explored further with a post hoc test for closer examination. The statistical analysis indicated a clear trend of significantly higher values of ADG for Ga compared to Hi throughout the experimental interval. The average total gain for both genders was 582.18 g for Hi and 664.49 g for Ga (Table 4).

### 3.2. Comparison with National Registry for Breed

A comparison of the experimental values obtained for the two breeds in the conditions of Cojocna Farm (Cluj, Romania) with the National Registry of Breed values registered at the national level in Romania can reveal how well these breeds adapted and how well they perform locally in terms of weight and ADG. The results of the *t*-test indicated that Hi male calves and female calves performed equally well or significantly better than the national average. Also, Ga male calves performed similarly well or better than the national average. By comparison, Ga female calves showed non-significant differences compared to the national average and a slight decrease in value, suggesting that their performance can be optimized (Table 5).

Overall, the comparison with the national average values indicates that the two breeds showed good adaptability in local conditions.

### 3.3. Results on Feed Quality

The statistical analysis showed that between the pastures grazed by the two breeds, there were some differences regarding protein content. The hay used as fodder for both breeds during winter months was richer in cellulose (Table 6).

There is a difference between the natural pasture of the *Festuca rupicola* type and *Stipa capillata-Bothriochloa ischaemum* type and the hay in terms of quality indicators as follows: the hay is richer in crude fiber (CF), neutral detergent fiber (NDF), and acid detergent fiber (ADF) but lower in fat (F) and crude protein (CP) than the pasture.

The relationship between parameters and ADG was studied using Pearson’s correlation.

**Figure 2:**
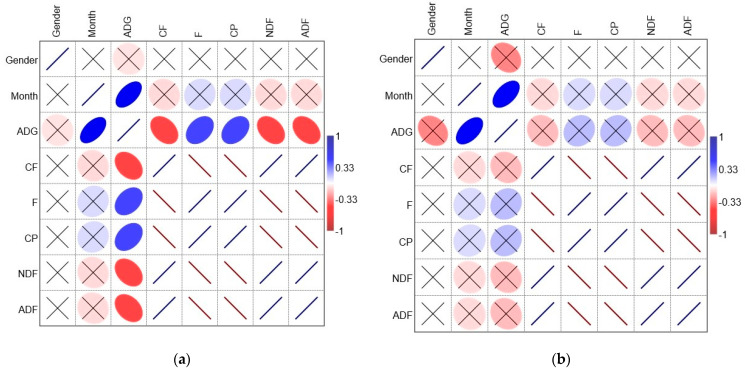
Relationship between variables: (**a**) Highland; (**b**) Galloway; ADG—average daily gain; CF—crude fiber; F—fat (crude); CP—crude protein; NDF—neutral detergent fiber; ADF—acid detergent fiber; Pearson’s correlation matrix, where size of ellipse is proportional to coefficient value, and crossed “×” indicates not significant at α 0.05.

A significant positive correlation was found between the month of measurements and ADG (Hi *r* = 0.56*, Ga *r* = 0.54*). A positive correlation was found between the raw fat (F) and protein (CP) of the feedstock and ADG. However, while the trend was similar, one can notice that these coefficients were significant only to Hi. At the same time, crude fiber (CF) and NDF and ADF correlate negatively with ADG. This suggests that fat and protein from the feedstock are significant to the ADG increase but more so to Hi, while the other feedstock parameters do not necessarily contribute to the ADG increase (Figure 2). 

## 4. Discussion

The Hi and Ga cattle breeds are among the oldest and purest native breeds in the world, raised exclusively for meat production, with high resistance to diseases, easy reproduction, high fertility, and an average lifespan of 20 years [20,21,22].

The animals of the Hi and Ga breeds are relatively small, compact, and robust, with a harmoniously developed physique. Their bodies are cylindrical with rounded shapes. The animals have a healthy skeletal structure and well-developed muscles, with strong legs and hooves. The most noticeable characteristic of these breeds is their long, two-layered coat. The outer layer consists of longer, coarser hairs that protect the animals from wind and rain, while the second layer, providing insulation and waterproofing, is soft and fine, which enables them to survive harsh winters outdoors, as they are very resistant to climatic changes [11,23,24].

Ga and Hi cattle, known for their adaptability to low-input, extensive grazing systems, are under-researched in Romania. This study evaluates the performance of Ga and Hi cattle in extensive grazing systems with low to medium quality, focusing on average daily gain (ADG) and adaptability to low-input conditions. The breeds showed consistent weight gains during the growth period, with a slight decrease in ADG from November to February, particularly for males.

In our study and in the scientific literature, the birth weight of Hi calves is around 24 kg, with significant variations from 17 to 45 kg, while Ga calves are slightly larger, weighing between 28 and 35 kg, depending on the season of birth, as spring-born calves tend to grow faster due to better grazing and milk intake [11,24]. An average adult female of the Hi breed can weigh up to 450 kg and the average male up to 700 kg, while the average female of the Ga breed can weigh up to 550 kg and the average male up to 900 kg [11,12,23].

Limited space for eating and drinking can lead to weight loss due to competition, with stronger animals feeding first. The winter period differences were more pronounced for males than females, contributing to understanding their role in sustainable and climate-resilient livestock farming. A study at the Animal Research Centre tested 11 breeds for their ability to give birth and rear calves. Ga cattle had the highest weaning rate (95.5%) and calf survival rate (95.2%) and an extremely low calving difficulty rate (0.8%). Ga calves are energetic at birth and begin suckling colostrum immediately, helping them to survive the critical first days of life. Ga females have highly developed maternal instincts, are very nurturing to their calves, and rear viable calves at a young age. They produce high-quality milk and care for their calves under all conditions [12,25,26]. Genetics and selection are crucial, sometimes more so than nutrition, with desirable traits including easy births, small calves, and gradual weight gain. Early calf development, including activities such as playing with hay and starting to eat before one month, also plays a key role.

The weights recorded in this study for both breeds are consistent with data from the National Breed Registry and the literature and align closely with findings from studies in Poland and Bulgaria. In Poland, male Ga calves reached 182–204 kg and females 158–163 kg at 210 days, while in Bulgaria, Ga calves fed high-quality forage weaned at 250–280 kg. Similarly, the average body weight at 210 days for Hi was 204 kg for males and 197 kg for females, while for Ga, males averaged 228.4 kg and females 203.7 kg [11,12,25,26]. Winter conditions, such as cold, rain, and snow, cause cattle to use more energy, halting or even reducing weight gain, as they focus on survival [27,28,29]. Feed efficiency varies due to factors like maintenance requirements, body condition, activity level, and digestive efficiency [30,31,32]. A study found that crossbred males required 15% more maintenance energy than Ga males [33,34]. The weight gains observed in this experiment, including during winter feeding on hay without supplementation, align with the literature, showing daily gains of 550 g for female Hi and 650 g for male Hi calves [35], and similar growth rates were seen in alpine pastures with average gains of 0.69 kg/day in the first year [24]. The Ga breed excels in breeding, requiring about 25% less concentrate and almost 10 kg less silage daily compared to other beef breeds. On natural pastures, indiscriminate grazing by Ga cows helps improve grasslands for livestock, wildlife, and game by removing excess roughage [12,36,37]. Ga has also become increasingly popular for controlling weeds and grasses with low forage value.

Natural grass is a vital nutrient source for ruminants due to its high digestibility and palatability, and it is widely used because it is economical. In Germany, Ga and Hi are preferred breeds due to their minimal environmental impact compared to other beef breeds, owing to their non-selective grazing habits and ability to travel long distances for food. Protein and water-soluble carbohydrates are key to evaluating forage nutritional value. Excessive protein content in early-maturing pastures can lead to high rumen nitrogen losses and low nitrogen utilization by grazing animals if the carbohydrate content is too low to balance it [38,39]. Many floristic studies have been conducted on the types of grasslands grazed by Hi and Ga, but forage quality remains under-researched.

From an agronomic perspective, the *Festuca rupicola* grassland is moderately tolerant to grazing and trampling, supporting a load of 0.61–0.80 L.U/ha under optimal conditions, while in areas with high woody vegetation and harmful species, the load can be as low as 0.01–0.20 L.U/ha. The *Stipa capillata—Bothriochloa ischaemum* grassland, which is more degraded, supports a load of 0.21–0.40 L.U/ha [17,40]. In Romania, the *Festuca rupicola* species is predominantly used for grazing, especially by sheep, and its forage value is highly dependent on pedoclimatic conditions and management practices [41,42,43].

### Limitations of This Study

This study acknowledges several limitations that could affect its statistical robustness and generalization. The first one is the small sample size of 16 animals (8 Hi and 8 Ga calves) and the focus on a single geographical region. However, this limitation is justified by the inherently small global populations of Hi and Ga cattle. Hi cattle, in particular, were declared at risk of extinction in the United Kingdom in 2021, underlining their limited availability worldwide. Moreover, these breeds are typically managed in small herds within extensive systems, contrasting with the large-scale, intensive operations associated with more common cattle breeds. To address these limitations, we compared the average daily gain (ADG) results recorded in this study with data from the National Breed Register, which encompasses information from 41 counties under diverse feeding regimes and geographical conditions from Romania (as described in the previous sections of this manuscript). The observed alignment between our findings and the national data strengthens the validity of our results, indicating that the performance trends observed are representative of these breeds under local conditions.

Considering these limitations and the current lack of studies on these two breeds, future research should focus on expanding the research on these two breeds, including larger sample sizes and incorporating multiple regions to provide a more comprehensive understanding of the adaptability and performance of Hi and Ga cattle. Such studies would contribute to refining their role within sustainable and climate-resilient livestock systems, offering valuable insights into optimizing management practices for these breeds in varied ecological settings.

## 5. Conclusions

Our findings demonstrate that Ga and Hi cattle effectively utilized the limited resources available in the study area, showing strong adaptability and satisfactory performance. Both breeds achieved a growth rate of approximately 600 g/day. Ga cattle, particularly male calves, outperformed Hi cattle; however, female calves exhibited more consistent performance during winter feeding with hay. When comparing weight and ADG to national averages, both breeds showed good adaptability and performance under local conditions. To the best of our knowledge, this study is one of the few conducted in Eastern Europe on these breeds, underscoring their potential to contribute to sustainable livestock farming in local environments. These findings suggest that Ga and Hi cattle could be key contributors to developing climate-resilient and sustainable livestock systems, particularly in regions with limited resources and challenging environmental conditions similar to those from the study area. As mentioned in the previous section, given this study’s limitations coupled with the current lack of studies on these two breeds, future research should focus on the following: (1) increasing the number of studies involving these two breeds; (2) expanding the sample size; and (3) including multiple regions with various feeding and climatic conditions.

## Figures and Tables

**Table 1:** Influence of factors and their interaction on parameters (*p*-values).

Feed	Date	Parameter	Breed (B)	Gender (G)	Interaction (B × G)
Suckle colostrum	At birth	Weight	0.0006 ***	0.0029 **	0.5381 ^n.s.^
Suckle milk + Grazing	IX 2023	Weight	0.0190 *	0.0171 *	0.1480 ^n.s.^
		ADG	0.0387 *	0.4307 ^n.s.^	0.8602 ^n.s.^
Hay	XI 2023	Weight	0.0032 **	0.0056 **	0.0194 *
		ADG	0.0364 *	0.3533 ^n.s.^	0.4667 ^n.s.^
Hay	II 2024	Weight	0.0007 ***	0.6649 ^n.s.^	0.0515 ^n.s.^
		ADG	0.0037 **	0.6807 ^n.s.^	0.3392 ^n.s.^
Grazing	V 2024	Weight	0.0040 **	0.0027 **	0.7955 ^n.s.^
		ADG	0.0073 **	0.1207 ^n.s.^	0.6321 ^n.s.^
-	Overall	TAG	0.0180 *	0.6108 ^n.s.^	0.5473 ^n.s.^

Note: measurements at birth—first day of life; ADG—average daily gain; IX 2023—September—weaning calves 210 ± 45 days of life—grazing; XI 2023—November fed on hay; II 2024—February fed on hay; V 2024—May—grazing; *p*-values from table according to two-way ANOVA; significance levels *p* > 0.05 (n.s.), *p* < 0.05 (*), *p* < 0.01 (**), *p* < 0.001 (***).

**Table 2:** The influence of experimental variants on weight measurements (kg).

Feed	Date	Breed	Gender	Mean	±SD	±SEM
Suckle colostrum	At birth	Hi	M	25.60 ^b^	1.34	0.60
			F	23.00 ^b^	1.00	0.58
		Ga	M	30.00 ^a^	2.12	0.95
			F	26.33 ^b^	1.53	0.88
Suckle milk + Grazing	IX 2023	Hi	M	204.00 ^b^	6.89	3.08
			F	197.00 ^b^	8.54	4.93
		Ga	M	228.40 ^a^	14.26	6.38
			F	203.67 ^b^	12.86	7.42
Hay	XI 2023	Hi	M	232.60 ^b^	4.93	2.20
			F	228.67 ^b^	2.08	1.20
		Ga	M	269.80 ^a^	17.63	7.88
			F	234.33 ^b^	9.71	5.61
Hay	II 2024	Hi	M	244.00 ^b^	6.20	2.77
			F	257.67 ^b^	7.02	4.06
		Ga	M	297.40 ^a^	24.83	11.10
			F	276.67 ^ab^	8.08	4.67
Grazing	V 2024	Hi	M	341.40 ^b^	10.06	4.50
			F	319.33 ^b^	5.86	3.38
		Ga	M	365.40 ^a^	15.50	6.93
			F	340.00 ^b^	13.11	7.57

Note: Hi—Highland; Ga—Galloway; M—male calves; F—female calves; measurements at birth—first day of life; IX 2023—September—weaning calves 210 ± 45 days of life—grazing; XI 2023—November fed on hay; II 2024—February fed on hay; V 2024—May—grazing; differences between means followed by at least one similar letter are not significant at α 0.05, according to Tukey’s test applied for each date.

**Table 3:** The influence of experimental variants on the average daily gain (ADG g).

Feed	Date	Breed	Gender	Mean	±SD	±SEM
Suckle milk + Grazing	ADG IX 2023	Hi	M	635.63 ^a^	87.70	39.22
			F	613.71 ^a^	32.57	18.81
		Ga	M	721.88 ^a^	70.98	31.74
			F	687.55 ^a^	15.77	9.11
Hay	ADG XI 2023	Hi	M	606.74 ^a^	70.69	31.61
			F	599.16 ^a^	33.79	19.51
		Ga	M	716.85 ^a^	79.00	35.33
			F	655.97 ^a^	68.25	39.40
Hay	ADG II 2024	Hi	M	501.05 ^b^	47.39	21.19
			F	538.84 ^ab^	24.83	14.33
		Ga	M	623.56 ^a^	71.20	31.84
			F	608.26 ^ab^	27.36	15.80
Grazing	ADG V 2024	Hi	M	599.38 ^ab^	36.85	16.48
			F	563.03 ^b^	28.72	16.58
		Ga	M	645.36 ^a^	29.02	12.98
			F	625.53 ^ab^	33.72	19.47

Note: Hi—Highland; Ga—Galloway; M—male calves; F—female calves; measurements at birth—first day of life; ADG—average daily gain; IX 2023—September—weaning calves 210 ± 45 days of life—grazing; XI 2023—November fed on hay; II 2024—February fed on hay; V 2024—May—grazing; differences between means followed by at least one similar letter are not significant at α 0.05, according to Tukey’s test.

**Table 4:** The influence of breed on average daily gain (ADG g).

Feed	Date	Breed	Mean	±SD	±SEM	Variance (*s*^2^)
Suckle milk + Grazing	ADG IX 2023	Hi	627.41 ^b^	69.47	24.56	4826.61
		Ga	709.01 ^a^	57.15	20.21	3266.03
Hay	ADG XI 2023	Hi	603.90 ^b^	56.54	19.99	3197.29
		Ga	694.02 ^a^	76.74	27.13	5889.24
Hay	ADG II 2024	Hi	515.22 ^b^	42.92	15.17	1841.93
		Ga	617.82 ^a^	56.34	19.92	3173.77
Grazing	ADG V 2024	Hi	585.75 ^b^	36.95	13.07	1365.62
		Ga	637.92 ^a^	30.19	10.67	911.51

Note: Hi—Highland; Ga—Galloway; measurements at birth—first day of life; ADG—average daily gain; IX 2023—September—weaning calves 210 ± 45 days of life—grazing; XI 2023—November fed on hay; II 2024—February fed on hay; V 2024—May—grazing; differences between pairwise means followed by at least one similar letter are not significant at α 0.05, according to Tukey’s test.

**Table 5:** Comparison of experimental values (Cojocna Farm) with National Breed Register mean values for Romania.

Factors		Parameter	Birth	Age (210 ±45 Days) IX 2023		Age (365 ±45 Days) XI 2023	
Breed	Gender		Weight kg	Weight kg	ADG g	Weight kg	ADG g
Hi	M	NRB (*n* = 138):	23.00	131.00	609.00	239.00	592.00
		Experimental:	25.60	204.00	636.63	232.60	606.74
		*t*:	2.75	23.68	0.69	-2.90	0.47
		*p*-value	0.0514 ^n.s.^	0.0000 ***	0.5305 ^n.s.^	0.0440 *	0.6635 ^n.s.^
	F	NRB (*n* = 238):	23.00	149.00	600.00	207.00	504.00
		Experimental:	23.00	197.00	613.71	228.67	599.16
		*t*:	0.00	9.73	0.73	18.03	4.83
		*p*-value	1.000 ^n.s.^	0.0104 *	0.54435 ^n.s.^	0.0031 **	0.0402 *
Ga	M	NRB (*n* = 119):	26.00	189.00	715.00	247.00	652.00
		Experimental:	30.00	228.40	721.88	269.80	716.85
		*t*:	4.22	6.18	0.21	2.89	1.84
		*p*-value	0.0135 *	0.0035 **	0.8411 ^n.s.^	0.0445 *	0.1400 ^n.s.^
	F	NRB (*n* = 250):	26.00	181.00	698.00	240.00	609.00
		Experimental:	26.33	203.67	687.55	234.33	655.97
		*t*:	0.38	3.05	−1.18	−1.01	1.19
		*p*-value	0.7418 ^n.s.^	0.0926 ^n.s.^	0.3587 ^n.s.^	0.4186 ^n.s.^	0.3565 ^n.s.^

Note: Hi—Highland; Ga—Galloway; M—male calves; F—female calves; ADG—average daily gain; IX 2023—September—weaning calves 210 ± 45 days of life—grazing; XI 2023—November 365 ±45 days—fed on hay; significance of *p*-values of *t*-test assigned according to thresholds *p* > 0.05 (n.s.), *p* < 0.05 (*), *p* < 0.01 (**), *p* < 0.001 (***).

**Table 6:** Pasture and hay—feed quality (mean ± SD).

Plant Material	CF	F	CP	NDF	ADF
Pasture (grass quality)	33.15 ± 3.16	3.50 ± 0.35	10.10 ± 1.89	57.54 ± 5.93	35.42 ± 2.14
Hay (winter fodder)	38.86 ± 1.11	2.82 ± 0.31	7.50 ± 0.51	65.16 ± 1.43	41.88 ± 1.27

Note: CF—crude fiber; F—fat (crude); CP—crude protein; NDF—neutral detergent fiber; ADF—acid detergent fiber.

## Data Availability

Data are contained within the article.

## References

[1] Greenwood P.L. (2021). Review: An overview of beef production from pasture and feedlot globally, as demand for beef and the need for sustainable practices increase. Animal.

[2] Qin Y., Kong H., Clark C., Lomax S., Su D., Eiffert S., Sukkarieh S. (2021). Intelligent perception for cattle monitoring: A review for cattle identification, body condition score evaluation, and weight estimation. Comput. Electron. Agric..

[3] (2020). FAOSTAT Faostat. http://www.fao.org/faostat/.

[4] Dillon J.A., Stackhouse-Lawson K.R., Thoma G.J., Gunter S.A., Rotz C.A. (2021). Current state of enteric methane and the carbon footprint of beef and dairy cattle in the United States. Anim. Front..

[5] Godde C.M., Mason-D’Croz D., Mayberry D.E., Thornton P.K., Herrero M. (2021). Impacts of climate change on the livestock food supply chain; a review of the evidence. Glob. Food Sec..

[6] Clay N., Garnett T., Lorimer J. (2020). Dairy intensification: Drivers, impacts and alternatives. Ambio.

[7] Phocas F., Belloc C., Bidanel J., Delaby L., Dourmad J.Y., Dumont B., Ezanno P., Fortun-Lamothe L., Foucras G., Frappat B. (2016). Review: Towards the agroecological management of ruminants, pigs and poultry through the development of sustainable breeding programs: I. selection goals and criteria. Animal.

[8] Cole J.B., VanRaden P.M. (2018). Symposium review: Possibilities in an age of genomics: The future of selection indices. J. Dairy Sci..

[9] Capper J.L., Bauman D.E. (2013). The role of productivity in improving environmental sustainability of ruminant production systems. Annu. Rev. Anim. Biosci..

[10] Dos Santos M.P., Morais T.G., Domingos T., Teixeira R.F.M. (2024). Measuring and scoring socioeconomic and environmental performance of Mediterranean pasture-based beef farms. J. Clean. Prod..

[11] Radkowski A., Radkowska I., Bocianowski J., Cyplik A. (2022). Weight gain of Highland cattle depending on the share of perennial ryegrass (*LoLium perenne* L.) in the meadow sward. Ann. Anim. Sci..

[12] Yarkov D. (2022). Galloway breed in Bulgaria—Contribution to sustainable beef cattle breeding. Trakia J. Sci..

[13] Bagnato A., Oltenacu P.A. (1994). Phenotypice valuation of fertility traits and their association with milk production of Italian Friesian cattle. J. Dairy Sci..

[14] Festila I., Miresan V., Raducu C., Coroian A., Constantinescu R., Cocan D. (2011). Study of productive performances in a dairy cows population of Simmental Type breed. Bull. UASVM Anim. Sci. Biotechnol..

[15] American Galloway. https://americangalloway.com/history.

[16] Onaciu G., Jurco E. (2014). Ghid practice pentru creșterea bovinelor. Casa Cărții De Știință Rom. Cluj-Napoca.

[17] Ranta M., Păcurar F., Gheţe I. (2024). The adaptation of the Galloway breed in the climatic conditions of the Cojocna Farm. Rom. J. Grassl. Forage Crops.

[18] De Castro M.L., Priego-Capote F. (2010). Soxhlet extraction: Past and present panacea. J. Chromatogr. A.

[19] Dale L. (2011). Determinarea Calitătii Furajelor Prin Metode Destructive si Non-Destructive. Ph.D. Thesis.

[20] Pauler M., Isselstein J., Braunbeck T., Schneider M.K. (2019). Influence of Highland and production-oriented cattle breeds on pasture vegetation: A pairwise assessment across broad environmental gradients. Agric. Ecosyst. Environ..

[21] Przysucha T., Grodzki H., Gołębiewski M., Slósarz J., Piotrowski T. (2013). Characteristic of Scottish Highland. Cattle. Med. Weter..

[22] Przysucha T., Grodzki H., Gołębiewski M., Slósarz J. (2013). Evaluation of the performance of Scottish Highland beef cattle in Poland. Med. Weter..

[23] Grubbe O. (2011). Galloway: Faszination einer Rinderrasse. Publ. Der Dtsch. Natl. Bibliogr..

[24] Berry N.R., Jewell P.L., Sutter F., Edwards P.J., Kreuzer M. (2002). Selection, intake and excretion of nutrients by Scottish Highland suckler beef cows and calves, and Brown Swiss dairy cows in contrasting Alpine grazing systems. J. Agric. Sci..

[25] Stuart G., McNeil H. (2023). Galloway Australia Magazine.

[26] Panayotova M. (2005). An example of ecological beef cattle breeding—Almost year round free-range farming under natural conditions. Zhivotnovadstvo Plyus.

[27] Fengels J., Kraft H. (2019). Deutsches Galloway Journal. Bundesverband Deutscher Gallowayzüchter e.V. Druck. Mergard Lauterb. Jahrg..

[28] Fengels J., Kraft H. (2020). Deutsches Galloway Journal. Bundesverband Deutscher Gallowayzüchter e.V. Druck. Mergard Lauterb. Jahrg..

[29] Fengels J., Kraft H. (2022). Deutsches Galloway Journal. Bundesverband Deutscher Gallowayzüchter e.V. Druck. Mergard Lauterb. Jahrg..

[30] Archer J.A., Richardson E.C., Herd R.M., Arthur P.F. (1999). Potential for selection to improve efficiency of feed use in beef cattle: A review. Aust. J. Agric..

[31] Voigt J., Jentsch W., Kuhla S., Matthes H.D., Derno M. (2000). Rumen fermentation and retention time of the digesta in growing cattle ofthe breeds Black-White Dairy Cattle, Galloway, and Highland. Arch. Anim. Breed..

[32] Wallace R.J., McPherson C.A. (1987). Factors affecting the breakdown of bacterial protein in rumen fluid. Brit. J. Nutr..

[33] Ivan M., Neill L., Entz T. (2000). Ruminal fermentation and duodenal flow following progressive inoculations of fauna-free wethers with major individual species of ciliate protozoa or total fauna. J. Anim. Sci..

[34] Martz F.A., Assay K.H., Hilolerbrand E.S., Payne C.G., Vogt J.R. (1974). Digestibility and rate of passage by steers fed tall fescue, alfalfa and orchad grass hay in 18 and 32 °C ambient temperature. J. Anim. Sci..

[35] Jentsch W., Derno M., Matthes H.-D., Löhrke B., Kuhla S., Scholze H. (1995). Ergebnisse aus Stickstoff- und Energieumsatzmessungen an adaptiv differenten Rindern. Arch. Anim. Nutr..

[36] Borys B., Rozwadowska M. (2010). Szkockie bydło górskie w Polsce—Piękno i użyteczność. Wiadomości Zootech..

[37] Blair P.H. (2023). Galloway Journal. Official Publication of the Galloway Cattle Society.

[38] Delagarde R., Peyraud J.L., Delaby L., Faverdin P. (2000). Vertical distribution of biomass, chemical composition and pepsin-cellulase digestibility in a perennial ryegrass sward: Interaction with month of year, regrowth age and time of day. Anim. Feed. Sci. Technol..

[39] Rearte D.H., Murphy J.J. (2005). New insights into the nutritional value of grass. Utilisation of Grazed Grass in Temperate Animal Systems, Proceedings of the Satellite Workshop of the 20th International Grassland Congress, Dublin, Ireland, 26 June–1 July.

[40] Păcurar F., Rotar I., Vidican R., Vaida I. (2023). The ecological and agronomic study of some grasslands phytocenoses from the Site Natura 2000 Rosci 0238 Suatu-Cojocna-Crairît. Rom. J. Grassl. Forage Crops.

[41] Păcurar F., Rotar I., Vidican R., Vaida I., Mălinaș A., Stoian V. (2016). Ecological and Agronomical Value of *Festuca rupicola* Grasslands. Rom. J. Grassl. Forage Crops.

[42] Rotar I., Păcurar F., Vidican R., Pleșa A., Vaida I., Gaga I. (2021). *Festuca rupicola’s* grassland from Tureni -Cluj after grazing with sheeps. Rom. J. Grassl. Forage Crops.

[43] Gaga I., Rotar I., Păcurar F., Vaida I., Pleşa A. (2022). The influence of organic and mineral fertilization on the production of *Festuca rupicola* grasslands in the Transylvian Plain. Rom. J. Grassl. Forage Crops.

